# Ethnic variation in medial orbital wall anatomy and its implications for decompression surgery

**DOI:** 10.1186/s12886-021-02009-y

**Published:** 2021-07-29

**Authors:** Minhui Amy Chan, Farah Ibrahim, Arjunan Kumaran, Kailing Yong, Anita Sook Yee Chan, Sunny Shen

**Affiliations:** 1 Singapore National Eye Centre, Singapore, Singapore; 2 Singapore Eye Research Institute, Singapore, Singapore; 3 Ophthalmology and Visual Sciences Academic Clinical Program, SingHealth Duke-NUS Academic Medical Centre, Singapore, Singapore

**Keywords:** Ethmoid sinus, Maxillary sinus, Orbital decompression surgery, Ethnic variations

## Abstract

**Background:**

To describe the inter-ethnic variation in medial orbital wall anatomy between Chinese, Malay, Indian and Caucasian subjects.

**Methods:**

Single-centre, retrospective, Computed Tomography (CT)-based observational study. 20 subjects of each ethnicity, were matched for gender and laterality. We excluded subjects younger than 16 years and those with orbital pathology. OsiriX version 8.5.1 (Pixmeo., Switzerland) and DICOM image viewing software CARESTREAM Vue PACS (Carestream Health Inc., USA) were used to measure the ethmoidal sinus length, width and volume, medial orbital wall and floor angle and the relative position of the posterior ethmoid sinus to the posterior maxillary wall. Statistical analyses were performed using Statistical Package for Social Sciences version 25.0 (IBM, USA).

**Results:**

There were 12 males (60 %) in each group, with no significant difference in age (*p* = 0.334–0.994). The mean ethmoid sinus length in Chinese, Malay, Indian and Caucasian subjects, using the Chinese as reference, were 37.2, 36.9, 38.0 and 37.4mm, the mean width was 11.6, 10.5, 11.4 and 10.0mm (*p* = 0.020) and the mean ethmoid sinus volume were 3362, 3652, 3349 and 3898mm^3^ respectively. The mean medial orbital wall and floor angle was 135.0, 131.4, 131.0 and 136.8 degrees and the mean relative position of posterior ethmoid sinus to posterior maxillary wall were − 2.0, -0.2, -1.5 and 1.6mm (*p* = 0.003) respectively.

**Conclusions:**

No inter-ethnic variation was found in decompressible ethmoid sinus volume. Caucasians had their posterior maxillary sinus wall anterior to their posterior ethmoidal walls unlike the Chinese, Malay and Indians. Awareness of ethnic variation is essential for safe orbital decompression.

## Background

Orbital decompression surgery is widely used in the management of severe dysthyroid optic neuropathy (DON) [1]. Other indications include disfiguring exophthalmos, exposure keratopathy from other causes of proptosis and retrobulbar pain [2]. Although many decompression techniques have been described: removing orbital bone or orbital fat or a combination of both, posterior medial orbital wall and posterior orbital floor decompression is still the preferred techniques for relieving apical compression in DON [3].

Medial orbital wall decompression was first described by Sewall in 1936 [4]. Further modifications were made by Walsh and Ogura via a transantral approach for combined medial wall and floor decompression in 1957 [5], and by Kennedy, endoscopically in 1985 [6]. Recent evolution involves sparing of the anterior medial orbital strut during decompression to minimize post-operative diplopia [7].

Traditionally, Lynch incision was used to access the medial orbital wall, however that resulted in significant post-operative scarring [8]. The more recent retrocaruncular approach provides the same exposure as the Lynch incision whilst avoiding the associated scarring [9]. It also allows maximal bone removal near the orbital apex with a more direct exposure of the medial extraperiosteal space [10].

Unlike the extensive literature on surgical techniques, there remains little data on the anatomical variations of the medial orbital wall despite their recognition clinically [11]. The few cadaveric [12, 13], dry skull [14, 15] and radiological-based studies [16, 17] on medial orbital wall anatomy, have not covered any inter-ethnic variation. Understanding of ethnic variation in medial orbital wall anatomy would improve the safety and efficacy of apical decompression for oculoplastic surgeons and help to identify boundaries for medial wall decompression. Hence, this study sought to describe the medial orbital wall measurements in 80 patients, to evaluate possible inter-ethnic variation.

## Methods

### Study design

Eighty Computed Tomography (CT) orbit scans from our facial trauma database (SNEC Eye Clinic@CGH, Changi General Hospital, Singapore; 2004–2010) were included in the study. Twenty random subjects were selected from each of the four major races in our population: Chinese, Malay, Indian and Caucasian. All groups were matched for gender and laterality – with the inclusion of 12 males and 8 females per ethnic group.

We excluded subjects below 16 years of age (with no upper limit for age), patients with other orbital pathology (i.e. Graves orbitopathy, orbital tumours) and the sides with orbital fractures.

All patient identifiers were removed from the scan data during the study. Informed consent was waived by the SingHealth Institutional Review Board (reference number 2016/3134) as the project was a retrospective radiology review with no patient contact and no patient identifiers. This study complied with the tenets of the Declaration of Helsinki and was approved by the SingHealth Institutional Review Board.

### Orbital Measurements

OsiriX version 8.5.1 (Pixmeo., Switzerland) and DICOM image viewing software CARESTREAM Vue PACS (Carestream Health Inc., USA) were used to measure the ethmoid sinus length, width and volume, the medial orbital wall and floor angle and the relation between the ethmoid and maxillary sinuses. All CT orbit scans were performed by Changi General Hospital’s Radiology department using the same standard imaging protocol of 1mm cut slices.

#### Ethmoid sinus length

**Fig. 1:**
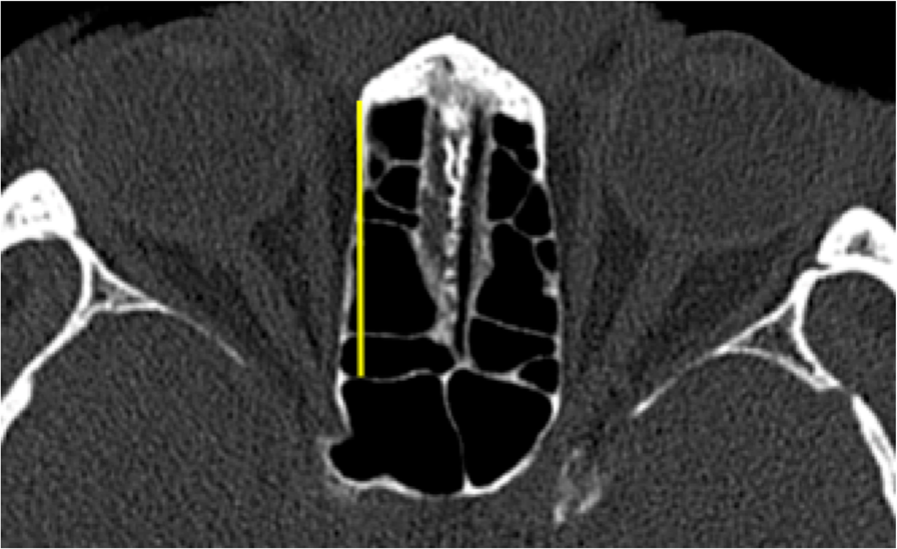
Ethmoid sinus length

On the axial view, the CT slide involving the superior most portion of the optic canal was selected, and the length of the ethmoid sinus was measured (yellow line) (Fig. 1).

#### Ethmoid sinus width

**Fig. 2:**
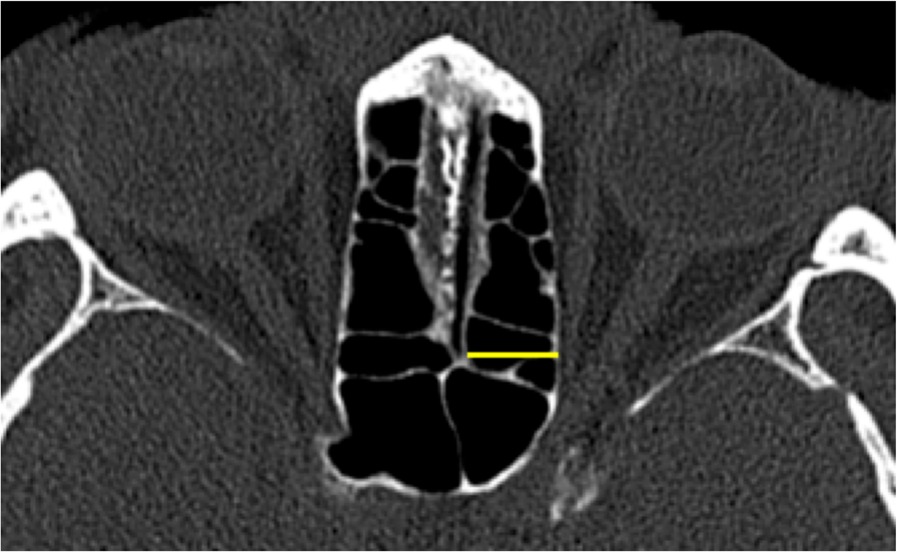
Ethmoid sinus width

On the same axial slide, the largest width of the ethmoid sinus was measured (yellow line) (Fig. 2).

#### Ethmoid sinus volume

**Fig. 3:**
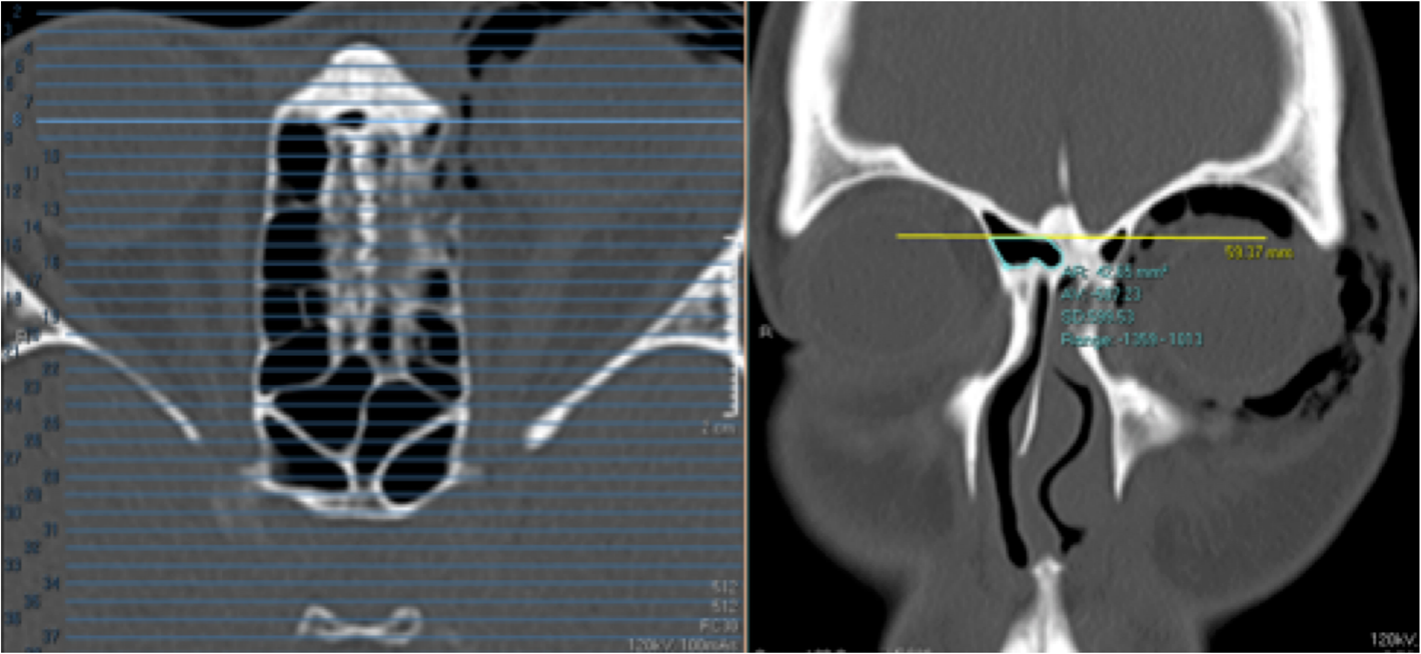
Ethmoid sinus volume – anterior limit. On the axial view (left image), anterior limit is set at level of posterior lacrimal crest. On the corresponding coronal view (right image), region of interest (ROI) tool is used to mark out the ethmoid sinus volume

**Fig. 4:**
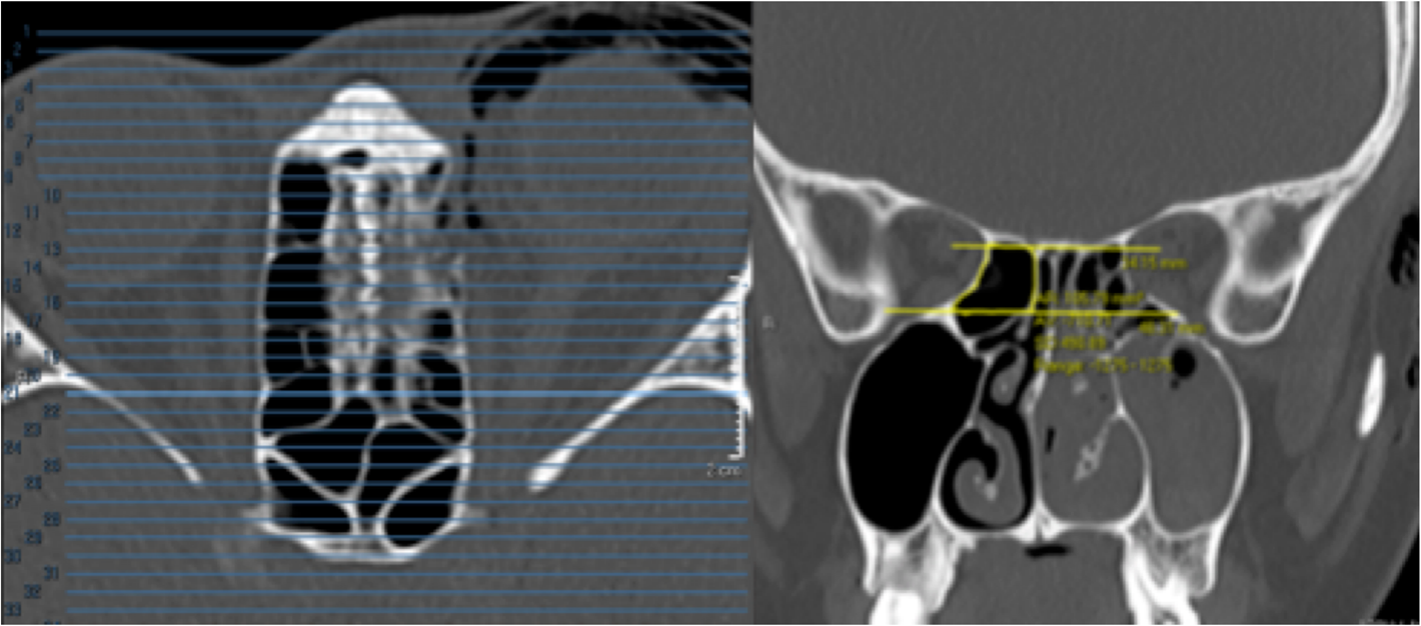
Ethmoid sinus volume – posterior limit. On the axial view (left image), the posterior limit is set at the anterior face of sphenoid sinus. On the corresponding coronal view (right image), ROI tool is used to mark out the ethmoid sinus volume

**Fig. 5:**
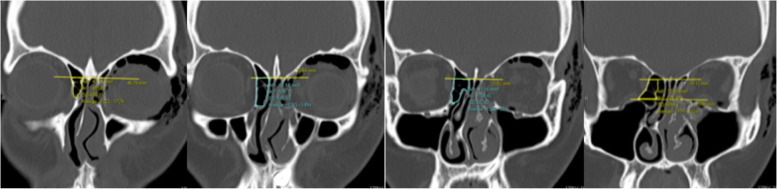
Ethmoid sinus volume – superior and inferior limits. The superior limit is set at the level of cribriform plate and the inferior limit is set at the intersection between medial orbital wall and orbital floor. Ethmoid sinus volumes are measured from anterior to posterior, with superior and inferior limits in place, and are added up to give the total volume (images below)

On both the axial and coronal views, the perimeter of the ethmoid sinus was marked with the Region of Interest (ROI) tool. The anterior limit for ethmoid sinus volume measurement was set at the posterior lacrimal crest and the posterior limit at the anterior face of the sphenoid sinus; the superior limit at the cribriform plate and the inferior limit at the intersection between the medial orbital wall and the orbital floor. The area of the marked segments (volume marked out) were added up to give the total ethmoid sinus volume (Figs. 3, 4 and 5). The ethmoid sinus volume is representative of the maximum decompressible volume during medial orbital wall decompression surgery.

#### Medial orbital wall and floor angle

**Fig. 6:**
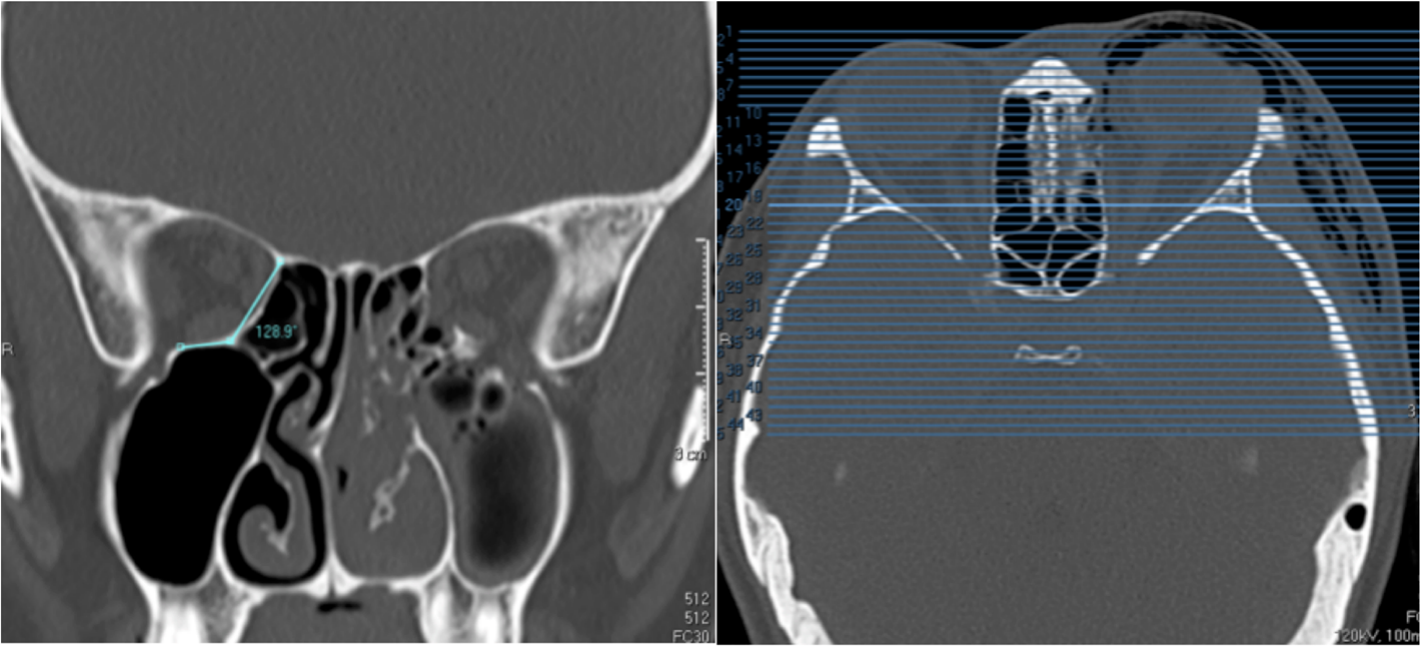
Medial orbital wall and floor angle. Measured on the coronal view (left image), at the level of posterior cortical bone of the trigones (right image)

The medial orbital wall and floor angle was measured on the coronal view, at the level of the posterior cortex of the bilateral trigone. The angle was formed between two lines, one along the medial orbital wall and the other along the orbital floor (Fig. 6).

#### Olfactory fossa depth

**Fig. 7:**
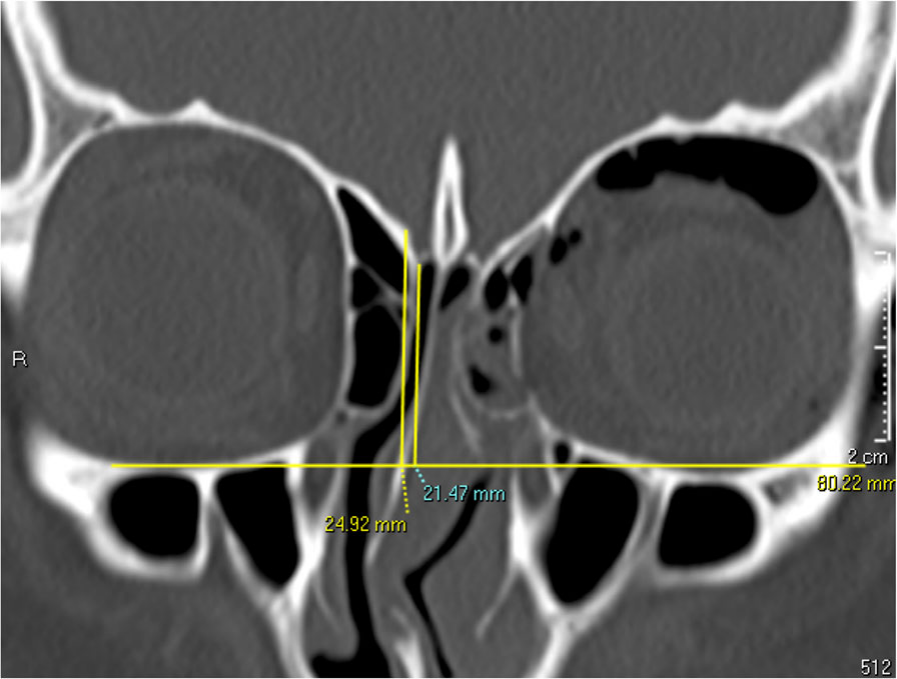
Olfactory fossa depth. Measured at slide where cribriform plate is deepest, taking the difference between the vertical length to the ethmoid roof and the vertical length to the cribriform plate from a horizontal line connecting both infraorbital nerves

Using the slide where the cribriform plate is deepest on the coronal view, a horizontal line is drawn connecting both infraorbital nerves. A vertical line is then drawn from the junction of the ethmoid roof to the lateral lamella of the cribriform plate (LLCP) to the marked horizontal line and a separate vertical line is drawn from the cribriform plate to the same horizontal line. The difference between the two vertical lines (yellow lines) signifies the vertical height of the lateral lamella of the cribriform plate (Fig. 7), which is then categorized using Keros’ classification. There are 3 Keros categories, namely Keros 1 (1-3mm), Keros 2 (4-7mm) and Keros 3 (8-16mm) [18].

#### Relative position of the posterior ethmoid sinus to posterior maxillary wall

**Fig. 8:**
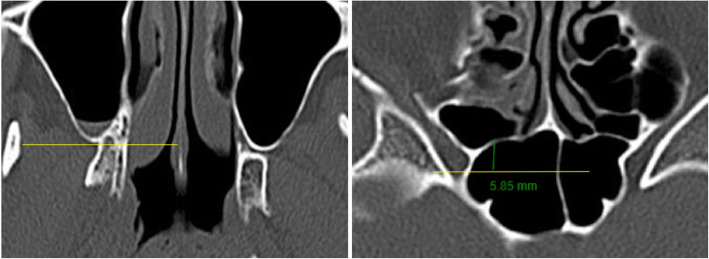
Relative position of posterior ethmoid sinus to posterior maxillary wall. Using the slide where the posterior maxillary wall is at its most posterior position, a horizontal line is drawn (yellow). A vertical line (green) is then drawn and measured from this horizontal line to the most posterior aspect of ethmoidal sinus. A positive value was given if the posterior maxillary wall lay anterior to the posterior ethmoidal wall. A negative value was given if the posterior maxillary sinus wall lay posterior to the posterior ethmoidal wall

On the axial series of scan, we first identified the slide where the posterior maxillary wall is at its most posterior and drew a horizontal line. Using the same technique, we also identified the most posterior of the ethmoid sinus. A vertical line was then drawn between these 2 horizontal lines. A positive value was given if the posterior maxillary walls lay anterior to the posterior ethmoid sinus. A negative value was given if the posterior maxillary wall lay posterior to the posterior ethmoid sinus (Fig. 8).

### Reliability indices

All measurements were done by the First author (AC) twice, 3 months apart, and again independently by the Second author (FI) to assess repeatability and reliability. Inter- and intra-observer variability was evaluated by Interclass coefficient correlations and Cronbach’s alpha.

### Statistical analysis

Data analysis was performed using Statistical Package for Social Sciences (SPSS) version 25 (IBM Corp., Armonk, NY). Descriptive analysis and comparisons between medial orbital wall measurements were evaluated. Two sample t-test was used for comparisons between groups with statistical significance set at *p* < 0.05.

## Results

A total of 80 orbit CT scans were included in our study. Each ethnic group (Chinese, Malay, Indian and Caucasian) had 20 subjects, of which 60 % were males (*n* = 12) and 50 % were right sided orbits (*n* = 10). There was no significant difference in the average ages between the groups (refer to Table 1).

**Table 1:** Demographics

	Chinese		Malays		Indians		Caucasians	
		*p*-value^#^		*p*-value^#^		*p*-value^#^		*p*-value^#^
Total number	20		20		20		20	
Males	12		12		12		12	
Laterality, right	10		10		10		10	
Minimum age, years	18		21		17		28	
Mean age, years (SD*)	42.4 (18.7)	-	42.3 (20.4)	0.994	42.7 (14.8)	0.948	47.4 (13.5)	0.334

*Standard deviation (SD)

^#^compared to Chinese subjects

The total mean ethmoid sinus length was 37.4mm (SD 3.7) and ranged from 27.0 to 45.8mm. The total mean ethmoid sinus width was 10.9mm (SD 2.0) and ranged from 7.5 to 16.1mm. We noted that the mean ethmoid sinus width was significantly wider in males (p 0.003) (11.4mm, SD 1.9) as compared to females (10.1mm, SD 1.9). There were otherwise no statistically significant differences noted with regard to ethmoid sinus length, volume and other orbital measurements between both genders (refer to Table 2).

**Table 2:** Gender specific and total mean medial orbital wall measurements

		Male			Female			Total	
		Mean	*p*-value^#^	Range	Mean	*p*-value^#^	Range	Mean	Range
Length, mm (SD*)		37.6 (3.4)	-	29.2–45.8	37.0 (4.1)	0.445	27.0–44.0	37.4 (3.7)	27.0-45.8
Width, mm (SD*)		11.4 (1.9)	-	8.2–15.5	10.1 (1.9)	**0.003**	7.5–16.1	10.9 (2.0)	7.5–16.1
Volume, mm^3^ (SD*)		3718 (862)	-	2120–5818	3336 (942)	0.065	1892–5267	3565 (909)	1892–5818
Medial orbital wall and floor angle, degrees (SD*)		134.6 (10.1)	-	116.1–163.0	131.9 (9.6)	0.236	109.6-149.1	133.5 (9.9)	109.6–163.0
Relative position of posterior ethmoid sinus to posterior maxillary wall, mm (SD*)		-0.2 (4.5)	-	-12.9-8.2	-1.0 (4.0)	0.398	-9.4-7.7	-0.5 (4.3)	-12.8-8.21

*Standard deviation (SD)

^#^compared to male subjects

Between the different racial groups, there was no statistically significant difference in the mean ethmoid sinus length. As compared to Chinese (11.6mm, SD 2.2), Caucasians had a narrower ethmoid sinus width (10.0mm SD 1.8, *p* = 0.020). There was otherwise no statistically significant difference in the ethmoid sinus width between Chinese, Malay and Indian subjects (refer to Table 3).

**Table 3:** Mean Ethmoid Sinus Length and Width

		Chinese		Malays		Indians		Caucasians	
Mean			*p*-value^#^		*p*-value^#^		*p*-value^#^		*p*-value^#^
Length, mm (SD*)		37.2 (4.6)	-	36.9 (1.0)	0.844	38.0 (3.0)	0.514	37.4 (0.7)	0.844
Width, mm (SD*)		11.6 (2.2)	-	10.5 (1.7)	0.086	11.4 (1.7)	0.711	10.0 (1.8)	**0.020**

*Standard deviation (SD)

^#^compared to Chinese subjects

**Fig. 9:**
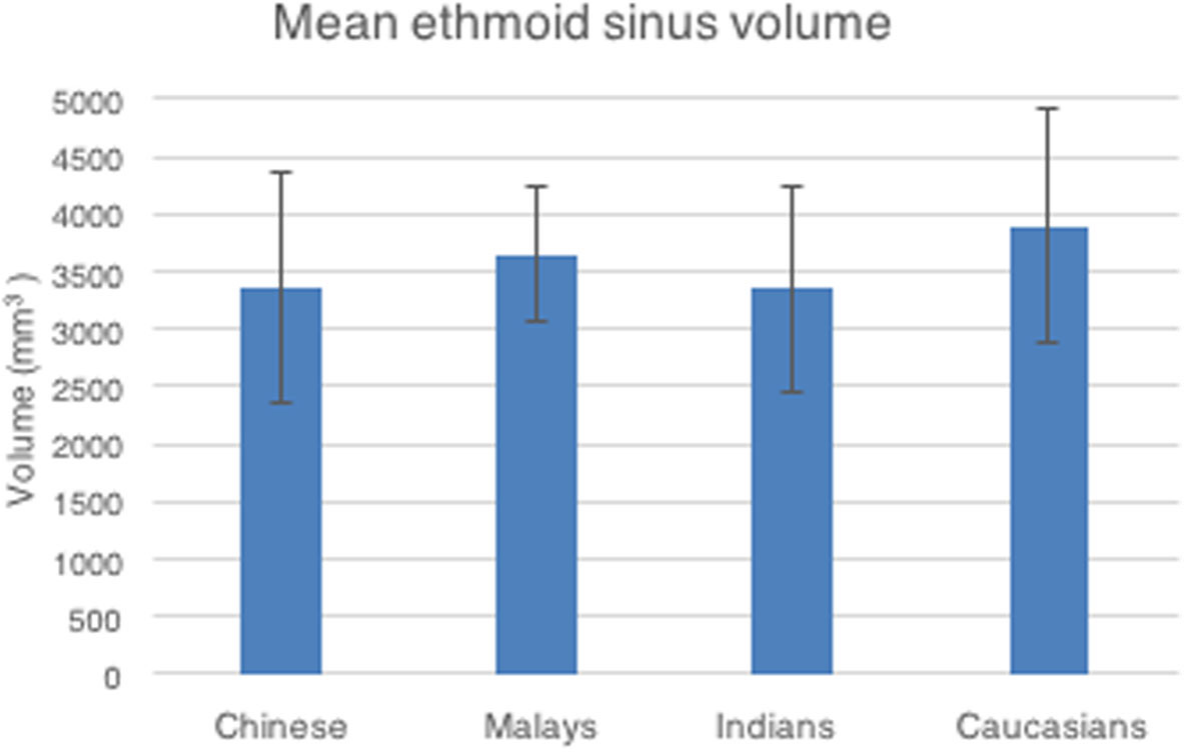
Mean ethmoid sinus volume

The mean ethmoid sinus volume was 3565mm^3^ (SD 909), with a range of 1892 to 5818mm^3^ (Table 2). However, amongst the racial groups, there was no statistically significant difference in the ethmoid sinus volume (*p*-value of 0.271; 0.964; 0.102) (Fig. 9).

**Fig. 10:**
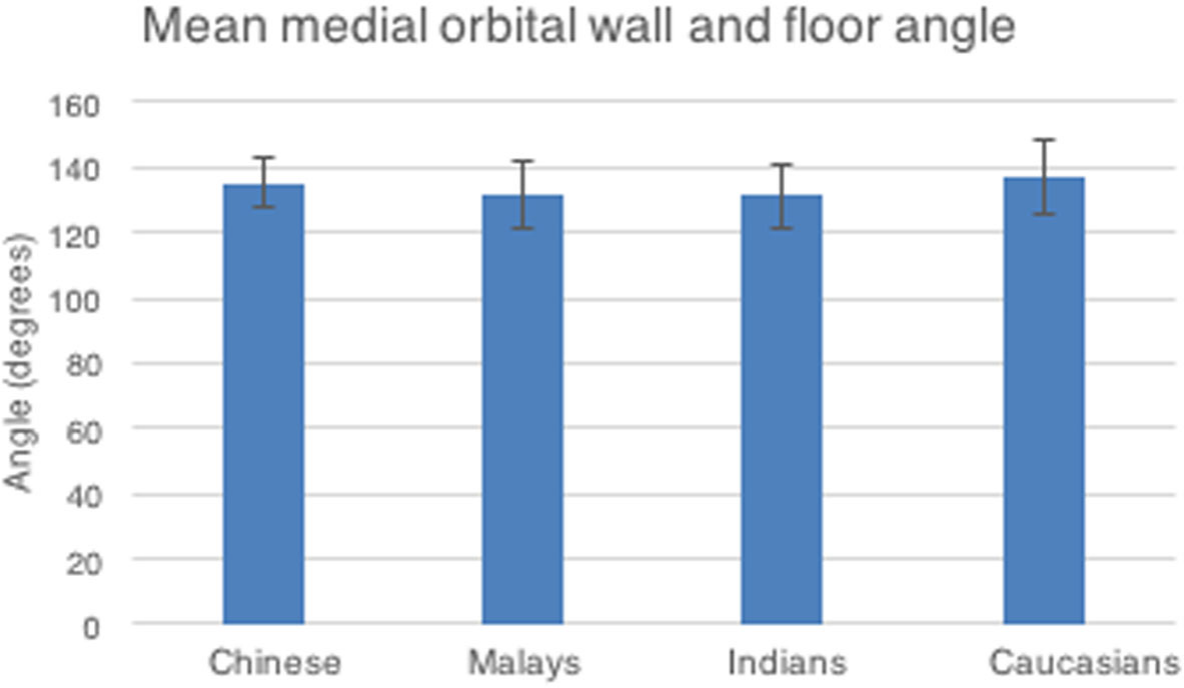
Mean medial orbital wall and floor angle

We noted the average medial orbital wall and floor angle to be 133.5 degrees (SD 9.9) with a range of 109.6 to 163.0 degrees (Table 2). Similarly, no ethnic variation was noted with regard to the medial orbital wall and floor angle (p-value of 0.223; 0.148; 0.560) (Fig. 10).

There were significantly more Caucasians (*p*-value 0.010) with shorter olfactory fossae depths compared to the Chinese. 60 % of Caucasians had Keros 2 olfactory fossa depth. None of the subjects fulfilled the Keros 3 classification (refer to Table 4).

**Table 4:** Mean Keros length (vertical depth of olfactory fossa) and type

		Chinese		Malays		Indians		Caucasians	
Mean			*p*-value^#^		*p*-value^#^		*p*-value^#^		*p*-value^#^
Keros length, mm (SD*)		3.1 (0.8)	-	2.9 (0.7)	0.329	3.1 (0.8)	0.949	4.0 (1.2)	**0.010**
Keros 1		18	-	18	**-**	16	-	8	**-**
Keros 2		2	-	2	**-**	4	-	12	-
Keros 3		0	-	0	**-**	0	-	0	-

*Standard deviation (SD)

** Keros type 1: 1-3mm, Keros type 2: 4-7mm; Keros type 3: 8-16mm

^#^compared to Chinese subjects

With regard to the relative position of posterior ethmoid sinus to posterior maxillary sinus wall, in Caucasians, the posterior maxillary wall was anterior to the posterior ethmoid wall / anterior face of sphenoid (1.6mm, SD 3.3, p-value 0.003), whereas in Chinese, Malay and Indians the posterior maxillary wall was further back compared to the posterior ethmoid wall (Table 5).

**Table 5:** Mean relative position of posterior ethmoid sinus to posterior maxillary wall

		Chinese		Malays		Indians		Caucasians	
Mean			*p*-value^#^		*p*-value^#^		*p*-value^#^		*p*-value^#^
Difference in length, mm (SD*)		-2.0 (3.9)	-	-0.2 (3.1)	0.100	-1.5 (5.8)	0.730	1.6 (3.2)	**0.003**

*Standard deviation (SD)

^#^compared to Chinese subjects

Inter and intra-observer variability are summarized below in Table 6.

**Table 6:** Reliability Indices of all measurements

			Intraclass Correlation (95 % CI)	
		Cronbach’s alpha	Intra-user	Inter-user
Ethmoidal length Ethmoidal width		1.000 1.000	1.000 1.000	1.000 1.000
Ethmoidal volume		1.000	0.999	1.000
Medial orbital wall and floor angle		1.000	0.999	0.999
Relative position of posterior ethmoid sinus to posterior maxillary wall		0.988	0.983	0.976

## Discussion

In today’s cosmopolitan society, it is not uncommon to have patients from different ethnic backgrounds. Even though variation in orbital anatomy is well recognised clinically, published data on ethnic variation is limited.

In our earlier study on lateral orbital wall anatomy [19], we have demonstrated that Indians, and to a lesser extent Caucasians, have smaller lateral wall trigones as compared to Chinese and Malays. This explains why lateral orbital decompression is technically more difficult in Chinese and Malay patients and often requires powered instruments. Indians also have shallower orbits which may limit the efficacy of lateral orbital decompression alone in reducing proptosis.

For patients with DON or severe proptosis in which lateral orbital decompression alone is ineffective or insufficient, removal of the medial orbital wall and/or orbital floor is required. In this study, we evaluate the medial orbital wall anatomy and its ethnic variations.

We recorded ethmoid sinus lengths ranging from 29.2-45.8mm in males and 27.0-44.0mm in females (Table 2), which were comparable to the existing literature (38.8-42.5mm in males and 36.4-40.8mm in females) [20]. There was no statistically significant difference between the ethnic groups. As a surrogate for the medial orbital wall, the mean ethmoid sinus length is rather constant and provides a reliable estimate on how deep a surgeon needs to decompress to relieve the apical crowding.

For the ethmoid sinus width, we were unable to compare our results (total mean width 10.9mm, 7.5-16.1mm in females, 8.2-15.5mm in males) with the published literature (15.1-17.5mm in males, 13.4-16.0mm in females) due to differences in measurement methods [20]. Males were noted to have wider mean ethmoid sinuses than females in our study and in existing literature [20]. Additionally, Caucasians were noted to have narrower ethmoid sinuses in our study as compared to the Chinese.

With regard to ethmoid sinus volume, our average measurement was 3.6cm^3^ (3.7cm^3^ in males and 3.3cm^3^ in females) and was most similar to the total mean of 4.51cm^3^ found in a Korean population by Park et al. [17]. There were published data of bigger ethmoid sinus volumes of 5.5cm^3^ (females) and 6.3cm^3^ (males) in a Turkish population [16] and total mean volume of 5.5cm^3^ in a Spanish population [21]. However, our study focused on the clinically decompressible ethmoid sinus volume from a retrocarcuncular approach instead of total ethmoidal volume and thus had smaller measured volumes.

Although the coronal slide we chose for measuring the medial orbital wall and floor angle may seem anterior to the true orbital apex, our measuring technique reduces the impact of sphenoid sinus size and location, which occasionally forms the medial wall of the optic canal and is not routinely removed in decompression surgery. Our mean angle value of 133.5° was close to Kang et al.’s 136.88° which was derived from 276 Asian orbits [22]. It is also similar to the 122° measured by Keast et al. in 36 Polynesian and 119° in 144 Caucasians [23]. This relative consistency in medial orbital wall and floor angle, reassured us of a minimum 4 clock hours wide apical relief in adequate posterior decompression surgery.

The olfactory fossa depth and Keros classification, guides us with the extent of superior bone removal in medial wall decompression. In our study, we noted an inclination towards Keros 2 in Caucasians as compared to the Chinese, differing from Badia, et al., who found no significant differences in olfactory depth between 100 Caucasians and 100 Chinese subjects [24]. This discrepancy may be due to our small sample size. However, our results concurred with Alazzawai, et al., where no significant differences were found between the Chinese, Malay and Indians in their Malaysian population with 80 % Keros 1 classification in 300 subjects, with none fulfilling Keros 3 criteria [25]. In Keros 3 patients, their ethmoidal roofs lie significantly higher than the cribriform plate, and thus bear the greatest risk of inadvertent intracranial entry during medial wall decompression [26].

Before the age of fine-cut CT scan and image-guided surgery, age-old wisdom suggested that the posterior ethmoidal wall (i.e. anterior sphenoid face) is about 1 cm behind the posterior maxillary wall. To prevent accidental entry into the sphenoid sinus and damaging the carotid syphon, one must sound out the posterior maxillary wall as a guide to see how far back decompression along the medial wall is needed (i.e. removing all the posterior ethmoid sinus until the anterior sphenoid face). In our study, we noted only Caucasians had their mean posterior maxillary wall anterior to the posterior ethmoidal wall / anterior sphenoid face. To our knowledge, there is only one other study, with 11 cadavers, where the anterior face of the sphenoid was noted to be about 2-4mm more posterior than the posterior maxillary wall [27]. For the Chinese, Malays and Indians in our study, their posterior ethmoidal wall is anterior to the posterior maxillary wall. This finding is similar to another Korean radiological study [28] of 115 CT scans, albeit with different measurement methods. This knowledge of ethnic variation in relative position of anterior sphenoid face to posterior maxillary wall is important in defining the safe zone of decompression. In reality, the sphenoidal wall is often thicker than the posterior ethmoidal wall, surgeons should think twice if they find the posterior medial orbital wall more difficult to break during decompression and when they are about 3-4 cm beyond the posterior lacrimal crest (i.e. mean ethmoid length) or 3-4mm from the posterior maxillary wall.

There were a few limitations in our study. Firstly, the sample size was relatively small and could have benefited from a larger number of subjects to allow for more accurate results. For example, we noted that Caucasians had larger ethmoid sinus volumes as compared to the Chinese but it did not reach statistical significance.

Secondly, the different ethnic groups may not be a completely homogenous sample, as there might have been Malays of Arab or of mixed Arab heritage, Chinese of Northern and Chinese of Southern descent for example.

Lastly, ethmoidal sinus anatomy is highly complex, despite the various methods used to demarcate and measure its volume, there remains much difficulty and variability in defining the boundaries of these intricate air cells [29]. We had strict and easily recognizable limits for demarcating the ethmoid sinus area for calculation and ensured that measurements were repeated twice by one observer and repeated again by a second observer to safeguard the reproducibility of the ethmoid sinus volume. On analysis, we found good inter and intra-user agreement with all our medial orbital wall measurements.

The strengths of our study include building on the same platform as earlier study on lateral orbital wall anatomy. Besides providing ethnic specific anatomical data for 4 different races, we explored the potential inter-ethnic variations in orbital wall anatomy, for which there is a dearth of literature on.

Future research in this area could expand on increasing the sample size and by involving other ethnic groups. Alternatively, the focus could also be shifted towards soft tissues measurements in normal or patients with pre-existing DON. This would allow for potential discovery of inter-ethnic pathological changes.

## Conclusions

There was no statistically significant inter-ethnic difference in ethmoid sinus length, decompressible ethmoid sinus volume and medial orbital wall and floor angle. Caucasians were found to have smaller ethmoid sinus widths and had a more anteriorly located posterior maxillary wall relative to their posterior ethmoidal wall, unlike the Chinese, Malay and Indians. Better awareness of some anatomical consistencies and ethnic variation in orbital anatomy is essential for safe orbital decompression surgery.

## Data Availability

The dataset supporting the conclusions of this article is included within the article and its additional file.

## Abbreviations

CT: Computed tomography
DON: Dysthyroid optic neuropathy
ROI: Region of Interest
LLCP: Lateral lamella of the cribriform plate
SPSS: Statistical Package for Social Sciences
